# Does greater thermal plasticity facilitate range expansion of an invasive terrestrial anuran into higher latitudes?

**DOI:** 10.1093/conphys/cov010

**Published:** 2015-03-13

**Authors:** Hugh S Winwood-Smith, Lesley A Alton, Craig E Franklin, Craig R White

**Affiliations:** School of Biological Science, The University of Queensland, Brisbane, QLD 4072, Australia

**Keywords:** Aerobic scope, cane toad, invasive species, metabolic rate, *Rhinella marina*, thermal plasticity

## Abstract

Temperature has pervasive effects on physiological processes and is critical in setting species distribution limits. Since invading Australia, cane toads have spread rapidly across low latitudes, but slowly into higher latitudes. Low temperature is the likely factor limiting high-latitude advancement. Several previous attempts have been made to predict future cane toad distributions in Australia, but understanding the potential contribution of phenotypic plasticity and adaptation to future range expansion remains challenging. Previous research demonstrates the considerable thermal metabolic plasticity of the cane toad, but suggests limited thermal plasticity of locomotor performance. Additionally, the oxygen-limited thermal tolerance hypothesis predicts that reduced aerobic scope sets thermal limits for ectotherm performance. Metabolic plasticity, locomotor performance and aerobic scope are therefore predicted targets of natural selection as cane toads invade colder regions. We measured these traits at temperatures of 10, 15, 22.5 and 30°C in low- and high-latitude toads acclimated to 15 and 30°C, to test the hypothesis that cane toads have adapted to cooler temperatures. High-latitude toads show increased metabolic plasticity and higher resting metabolic rates at lower temperatures. Burst locomotor performance was worse for high-latitude toads. Other traits showed no regional differences. We conclude that increased metabolic plasticity may facilitate invasion into higher latitudes by maintaining critical physiological functions at lower temperatures.

## Teaser

In Australia Cane toads have invaded further into high latitude regions than expected. Comparison of high and low latitude toads reveals that high latitude toads have an increased ability to raise their metabolic rate when acclimated to low temperatures. This increased thermal plasticity may be facilitating the ongoing invasion into high latitude regions.

## Introduction

Recent evidence indicates that biodiversity is declining on a global scale (Butchart *et al.*, 2010). This is a major concern because of the largely negative impact that a reduction in species richness has on many ecosystem functions (Cardinale *et al.*, 2012). Human activity is a major cause of this reduction in species richness, primarily through actions leading to habitat loss (Hanski, 2011). The transportation of alien species into new environments also has the potential to impact biodiversity significantly and is a factor that has become increasingly relevant as human activity has globalized (Mooney and Cleland, 2001). Invasive species may reduce or eliminate native populations through processes such as predation (Savidge, 1987; Case and Douglas, 1991; Marsh and Douglas, 1997; Burbudge and Manly, 2002; Blackburn *et al.*, 2004), competition (Porter and Savignano, 1990; Kenward and Holm, 1993; Douglas *et al.*, 1994; Petren and Case, 1996; Holway, 1999; Byers, 2000) and pathogenicity (Mamaev *et al.*, 1995; Whittington *et al*., 1997; Wyatt *et al.*, 2008). Understanding the factors that allow a given alien species to invade successfully, i.e. to colonize and flourish in a new environment, is critical for their management, and numerous studies have been designed to improve our understanding of what factors predict invasive success (Gallien *et al.*, 2010). There are many hypotheses that attempt to explain determinants of successful invasion, implicating factors such as the traits of the invaders, size and frequency of introductions and the geography at the sites of introduction (reviewed by Catford *et al.*, 2009), but no consensus regarding what creates a successful invader or even how best to address this question experimentally has been reached (van Kleunen *et al.*, 2010). Instances of post-arrival adaptation of invaders have been demonstrated repeatedly, suggesting that local adaptation may be an important factor for invasive success, yet this is often not considered in studies aiming to determine what makes some species superior colonists (Whitney and Gabler, 2008). The present study investigates the possibility of local adaptation among populations of the highly invasive cane toad (*Rhinella marina*) within Australia, where this species has had a significant negative ecological impact. The question of whether adaptation will facilitate invasion beyond current predictions is critical for informing management strategies.

The cane toad (*R. marina*) is a large anuran species native to Central and South America (Zug and Zug, 1979) that has been introduced to numerous countries around the world, including Australia, Puerto Rico, Fiji and the USA (Hawaii and Florida), as a method of agricultural pest control (Easteal, 1981). The cane toad is toxic (Smith and Phillips, 2006; Hayes *et al.*, 2009), exhibits a flexible breeding cycle with high reproductive output (Lampo and Medialdea, 1996; Shine, 2010), has proved to be adept at colonizing new environments (Lever, 2001) and is considered to be one of the 100 worst invasive alien species in the world (Lowe *et al.*, 2000). In Australia, the cane toad has gained a significant foothold, spreading to >15% of the continent since its introduction in 1935 (Kolbe *et al.*, 2010). After its release into tropical Queensland, the cane toad expanded its range predominantly across lower latitudes in northern Australia and is now present in northern Western Australia.

How far introduced cane toads will ultimately spread has been a question that has commanded considerable attention, and several predictive studies have been carried out in an attempt to provide an answer (reviewed by Phillips *et al.*, 2008). The first of these suggested that cane toads had already exceeded their expected distribution limits in Australia (Sutherst *et al.*, 1996), but more recent studies all predict cane toad distributions that extend significantly further at lower latitudes than the present distribution and predict comparatively minimal expansion into higher latitudes (Urban *et al.*, 2007; Kearney *et al.*, 2008; Kolbe *et al.*, 2010). In addition, in all three of these recent studies the potential for adaptation to confound predictions of future distributions is discussed. This possibility deserves attention because species removed from their native environment may be afforded the opportunity to adapt in novel ways due to the addition and subtraction of various selection pressures (Ghalambor *et al.*, 2007; Sexton *et al.*, 2009).

Establishing evidence of local adaptation within populations is challenging. To demonstrate that phenotypic differences between populations have a genetic basis, multiple generations must be raised in common controlled conditions; the so-called ‘common garden experiment’ (Kawecki and Ebert, 2004). Such an experimental approach is necessary to account for the confounding effects of phenotypic and developmental plasticity (Wilson and Franklin, 2002). This approach has been used to demonstrate heritable differences in growth rate (Phillips, 2009) and dispersal rate (Phillips *et al.*, 2010) between populations of cane toads along the rapidly expanding northwest invasion front and long-established populations near the point of introduction. Adaptation of other traits, including limb morphology, hopping speed, endurance and desiccation resistance (Phillips *et al.*, 2006; Llewelyn *et al.*, 2010; Jessop *et al.*, 2013), has also been implicated as a mechanism facilitating the accelerating northwest push across low latitudes, but no study as yet has considered whether adaptation to cold conditions is facilitating the movement of cane toads into higher latitudes.

A recent theory that provides a mechanistic explanation for temperature limitation in ectotherms is the oxygen-limited thermal tolerance (OLTT) hypothesis (Zielinksi and Pörtner, 1996; De Watchter *et al.*, 1997). This hypothesis postulates that thermal limits (i.e. the minimal and maximal temperatures at which an organism can survive) in ectotherms are determined by the inability of the circulatory system to deliver oxygen above and beyond an organism's basal metabolic requirements at low and high temperatures. This difference between maximal oxygen supply and the metabolic cost of basic physiological maintenance (aerobic scope) allows an organism to devote energy to additional functions that enhance its Darwinian fitness, such as locomotion and reproduction (Bennett and Ruben, 1979; Carey, 1979). Several studies have provided support for the OLTT hypothesis among aquatic vertebrates and invertebrates (Frederich and Pörtner, 2000; Mark *et al.*, 2002; Peck *et al.*, 2002; Lannig *et al.*, 2004; Nilsson *et al.*, 2009; Neuheimer *et al.*, 2011; Verberk and Calosi, 2012), but relatively little attention has been given to its applicability for terrestrial species. Only two studies have tested OLTT on a terrestrial vertebrate ectotherm, both on the cane toad, but neither found any evidence for OLTT (Seebacher and Franklin, 2011; Overgaard *et al.*, 2012).

An interesting observation to emerge from the studies by Seebacher and Franklin (2011) and Overgaard *et al.* (2012) was that cane toads acclimated to 20 and 30°C showed no difference in metabolic rate when measured at their respective acclimation temperatures, despite a 10°C difference in test temperature, demonstrating the significant thermal metabolic plasticity of the species (Seebacher and Franklin, 2011; Overgaard *et al.*, 2012). Such a response suggests that maintaining metabolic rate above a minimal threshold may be an important strategy for coping with low temperatures; hence, metabolic plasticity may be key to expanding the cane toad's thermal niche. While increased metabolic rate may be considered a cost rather than a benefit, it is an energetic cost that can be met to facilitate a fitness benefit, e.g. the increased intake hypothesis (Nilsson, 2002), and there are studies demonstrating an association between increased metabolic rate and beneficial traits such as social dominance (Røskaft *et al.*, 1986; Hogstad, 1987; Yamamoto *et al.*, 1998; Reid *et al.*, 2011), reproductive success (Boratyński and Koteja, 2010) and over-winter survival (Jackson *et al.*, 2001; Boratynski *et al.*, 2010). Seebacher and Franklin (2011) also found that accumulation of blood lactate increased during exercise at low temperatures, indicating an increase in the energetic cost of locomotion. This increased expense was not alleviated by low-temperature acclimation. Likewise, Miller and Zoghby (1986) previously demonstrated a failure of acclimation at 12°C to improve locomotor performance at 12°C over cane toads acclimated at 22°C. Thus low-temperature constraints on locomotor capacity and performance may be limiting the ability of the cane toad to advance into colder regions.

In the present study, we investigated the possibility that cane toads on the high-latitude invasion front in Australia are adapting to colder environments, and thus have the capacity to advance further into high-latitude regions. This was done by measuring key physiological traits predicted to be limiting for the species at cold temperatures and thus possible targets for natural selection to facilitate dispersal into colder climates. Cane toads from high (southern) and low (northern) latitudes were acclimated for a minimum of 8 weeks to temperatures of 15 or 30°C. The resting rate of oxygen consumption (V.o_2_rest; a proxy for the metabolic cost of basic physiological maintenance), peak post-exercise rate of oxygen consumption (V.o_2_max; a measure of maximal oxygen supply capacity), aerobic scope (V.o_2_rest; subtracted from V.o_2_max) and locomotor performance were measured at 10, 15, 22.5 and 30°C to test the following hypotheses: (i) based on the considerable thermal metabolic plasticity of cane toads (Seebacher and Franklin, 2011; Overgaard *et al.*, 2012) and the associated implications for cold temperature tolerance, it is predicted that high-latitude toads will show a greater increase in metabolic rate than low-latitude toads when acclimated to cold temperatures; (ii) based on the predictions of Seebacher and Franklin (2011) that limited capacity to acclimate locomotor performance to cold temperatures may limit the spread of this species into colder climates, it is predicted that high-latitude toads will show enhanced locomotor performance at low temperatures compared with low-latitude toads; and (iii) based on the predictions of the OLTT hypothesis, high-latitude cane toads will show a greater aerobic scope than low-latitude cane toads at lower temperatures.

## Materials and methods

### Animal collection and maintenance

Low-latitude cane toads were collected from two populations in northern Queensland in September and October 2012. The first population was sourced from a 150 km stretch of coastline adjacent to the Gulf of Carpentaria, and the second was collected in Mareeba. These populations were chosen due to their proximity to Cairns, where cane toads were first introduced to Queensland. High-latitude cane toads were collected from two populations in Ballina and Yamba in northern New South Wales in December 2012 (Supplementary Fig. S1). New South Wales Parks and Wildlife officers specializing in invasive species management reported that these represent some of the most southern locations in Australia where cane toads are well established.

Once transported to The University of Queensland, 40 toads from each region (high and low latitude) were divided equally and randomly across two acclimation temperatures of 15 and 30°C. Toads were maintained in groups of five in plastic containers 65 cm long × 35 cm wide × 35 cm deep containing water and damp bark chips deep enough for burrowing. Containers were housed inside two temperature-controlled rooms, in which the acclimation temperatures were regulated to ±1.5°C, and maintained a 12 h light–12 h dark photoperiod. All toads were acclimated to these conditions for a minimum of 8 weeks and a maximum of 14 weeks before measurements. Animals were collected at different times during the year to reduce the time over which measurements were taken and to align with the later emergence of high-latitude toads, thus the collection and acclimation of high-latitude toads began as experimental procedures on low-latitude toads commenced. Toads maintained at 30°C were fed an average of four adult cockroaches (*Nauphoeta cinerea*) per week, while those at 15°C were fed two adult cockroaches per week. This was chosen based on the reported energy density of this cockroach (Secor and Faulkner, 2002) and cane toad resting rates of oxygen consumption determined in a previous study (Halsey and White, 2010).

### Physiological traits and test temperatures

The physiological traits of V.o_2_rest, V.o_2_max, aerobic scope and locomotor performance were measured at the four test temperatures of 10, 15, 22.5 and 30°C. The V.o_2_rest was measured during the inactive circadian phase (day). The V.o_2_max and locomotor performance were measured during the active circadian phase (night). A stratified random-order regimen was used for all measurements.

### Resting rate of oxygen consumption

The V.o_2_rest (in millilitres of O_2_ per hour) of an individual toad was measured using positive-pressure flow-through respirometry. Atmospheric air was drawn from outside using a pump (TR-SS3; Sable Systems, USA) and scrubbed of CO_2_ using soda lime and water vapour using Drierite before passing through a mass flow controller (GFC17; Aalborg, Orangeburg, NY, USA) that regulated the flow rate to a nominal value of 50 ml min^−1^ at 10, 15 and 22.5°C and 100 ml min^−1^ at 30°C. Mass flow controllers were calibrated using a NIST-traceable bubble film flowmeter (1–10–500 ml; Bubble-O-Meter, Dublin, OH, USA). After the mass flow controller, air was rehumidified using a 1 litre gas-washing bottle (Schott, French's Forest, NSW, Australia) before passing through the respirometry chamber (460 ml plastic container). The humidifying gas-washing bottle and respirometry chamber were housed inside a temperature-controlled cabinet (ERI140; ProSciTech, Thuringowa, QLD, Australia) that regulated the test temperature to ±0.5°C. After the respirometry chamber, air was scrubbed of water vapour using Drierite before passing through a CO_2_ analyser (LI-7000; LI-COR, Lincoln, NE, USA) and an O_2_ analyser (Oxzilla II; Sable Systems, Las Vegas, NE, USA). The CO_2_ and O_2_ analysers were interfaced with a PowerLab 8/30 A/D convertor (ADInstruments, Bella Vista, NSW, Australia), which recorded fractional concentrations of CO_2_ and O_2_ in the excurrent air at a frequency of 10 Hz. The CO_2_ analyser was calibrated with dry CO_2_-free air and a certified gas mixture (0.386 ± 0.008% CO_2_ in N_2_; BOC Gases, Wetherill Park, NSW, Australia), and the O_2_ analyser was calibrated using dry CO_2_-free air.

Before being measured, toads were fasted for a minimum of 5 days (Secor and Faulkner, 2002; Halsey and White, 2010). Measurements were conducted on a by-container basis, and the measurement order of the eight containers within a region (high or low latitude) was randomized. Blocks of four containers were tested at a given test temperature, and the order of the test temperatures was randomized. Each time a container was tested, the order in which the individuals within that container were tested was randomized. The four individuals that were to be tested on a given day were maintained in the temperature-controlled cabinet at the test temperature for the night prior to being measured. Respirometry measurements on individual toads were conducted for 4 h, with two toads being measured simultaneously in the morning and two toads measured simultaneously in the afternoon. The rate of oxygen uptake (V.o_2_) and rate of carbon dioxide production (V.o_2_) were calculated according to standard equations (Lighton, 2008) for the 3 h period with the lowest mean V.o_2_.

### Peak post-exercise rate of oxygen consumption

Logistical constraints dictated that the measurement regimen for V.o_2_max (in millilitres of O_2_ per hour) differed between high- and low-latitude toads. Low-latitude toads were tested on consecutive nights after all V.o_2_rest measurements were complete, with 20 toads measured per night at a single test temperature each night until all toads had been tested at all test temperatures. As with V.o_2_rest measurements, all toads in a container were tested consecutively in a random order, with the order of containers from both acclimation treatments also randomized. For high-latitude toads, every 5 days following a block of V.o_2_rest measurements on 20 toads at a given test temperature, those toads underwent the V.o_2_max measurements the following evening at the same test temperature and in the same random order in which they had undergone V.o_2_rest measurements. Both high- and low-latitude toads were placed in a temperature-controlled cabinet at the test temperature for the night prior to measurements in enclosures similar to those in which they normally lived.

To obtain estimates of V.o_2_max, toads were exercised in a swimming flume (SW10100; Loligo Systems, Tjele, Denmark) at a water velocity of 0.35 m s^−1^ until exhaustion, and then oxygen consumption was immediately measured using the same respirometry system described for V.o_2_rest measurements. Similar methods have previously been shown to provide values of V.o_2_ that approximate, if not exceed, V.o_2_max during exhaustive exercise and are thus a good indication of maximal respiratory capacity (Reidy *et al.*, 1995). Flow velocity was measured just below the surface of the water in the middle of the swim chamber (Hand-held unit HFA and 30 mm vane wheel probe, ZS30GFE-md20T/100/p10; Hontzsch, Waiblingen, Germany). The test temperature was controlled by circulating water from a temperature-controlled water bath (CB 8-30E, Heto, Allerød; Denmark) through the heat exchanger of the flume. Toads were encouraged to swim continually by gentle prodding with a wooden spatula. When toads stopped swimming and fell back against the rear of the chamber, their heads were submerged and they were encouraged to continue swimming with gentle prods from behind. If this was done four times in quick succession and the toads still refused to swim, they were considered to be exhausted and were removed from the swim chamber. If toads felt limp in hand they were immediately placed within the respirometry chamber, but if they struggled against being handled then they were returned to the swim chamber and encouraged to keep swimming. Once exhausted, toads were immediately placed within the respirometry chamber to measure V.o_2_max. At 15, 22.5 and 30°C, toads were left within the respirometry chamber for 5 min. At 10°C, toads had a tendency to hold their breath upon placement inside the respirometry chamber, so were left within the chamber until oxygen consumption was observed to rise and then for a further 5 min. The respirometry chamber was submerged within the water of the buffer tank of the swimming flume to regulate temperature. The mass flow controller in the respirometry system regulated the flow rate to a nominal value of 500 ml min^−1^ at all test temperatures.

Instantaneous rates of oxygen consumption were calculated using the method of Bartholomew *et al.* (1981). The effective chamber volume (including the chamber, tubing and analysers) was measured using nitrogen washout tests, with dead toads of different sizes placed within the chamber. There was no effect of toad mass on the measured effective volume, so a mean value calculated for the different-sized toads was used. Opening the respirometry chamber caused a transient 16 s decrease in O_2_ concentration associated with the admittance of room air; therefore, 16 s of data were removed from the beginning of each measurement. The highest value for oxygen consumption occurring during the remainder of the 5 min period was then used as an estimate of V.o_2_max.

### Locomotor performance

Measurements of both locomotor endurance and burst locomotor performance were made during the exercise phase that was used to obtain estimates of V.o_2_max. A camera (PS3eye; Sony Corporation, Japan) mounted above the swim chamber and connected to a PC was used to record each swimming session at 60 frames s^−1^ using VirtualDub v1.9.11 (www.virtualdub.org). The time for toads to reach exhaustion was used as a measurement of locomotor endurance. The largest distance that they were able to propel themselves with a single jump from the back of the chamber against the water current was used as a measurement of burst locomotor performance. Jumps were considered for analysis if the toad was not prodded or touched, did not push off from the sides and did not leave the water. Jump distance was measured from the video footage with Kinovea v0.8.15 (www.kinovea.org).

### Aerobic scope

Absolute aerobic scope was calculated by subtracting V.o_2_rest from V.o_2_max. Absolute aerobic scope was used as opposed to factorial aerobic scope (ratio of V.o_2_max to V.o_2_rest) because factorial scope was not considered to be informative (Clark *et al.*, 2013)_._

### Statistical analyses

Data for each physiological trait were analysed with a linear mixed-effects model using maximum likelihood using the lme4 package v1.0-4 (Bates *et al.*, 2013) of R v3.0.2 (R Core Team, 2013) in RStudio v0.97.551 (RStudio, 2013) with region (low latitude or high latitude), acclimation temperature (15 or 30°C), test temperature (10, 15, 22.5 or 30°C), body mass, time spent at the acclimation temperature and the full factorial combination of two- and three-way interactions among acclimation temperature, test temperature and region as fixed effects, with population (Gulf, Mareeba, Ballina and Yamba), container (nested within population) and individual identity (nested within container and population) as random effects. The significance of the random effects and the fixed effect of time spent at the acclimation temperature was examined first using likelihood ratio tests, and non-significant effects were removed from subsequent models. The significance of the remaining fixed effects within this minimum adequate model were then tested using likelihood ratio tests. Random effects were retained within the minimum adequate model if significant at α = 0.25 according to the recommendations of Quinn and Keough (2002), and α was set at 0.05 for tests of significance for the fixed effects. When the main effect(s) of region or acclimation temperature were significant or any interaction involving these effects was significant, *post hoc* pairwise comparisons were made among groups at each test temperature using linear models when the model included no random effects or likelihood ratio tests when the model did include random effects. For details of the results of tests involved in model simplification see the Supplementary material.

## Results

### Resting rate of oxygen consumption

**Figure 1:**
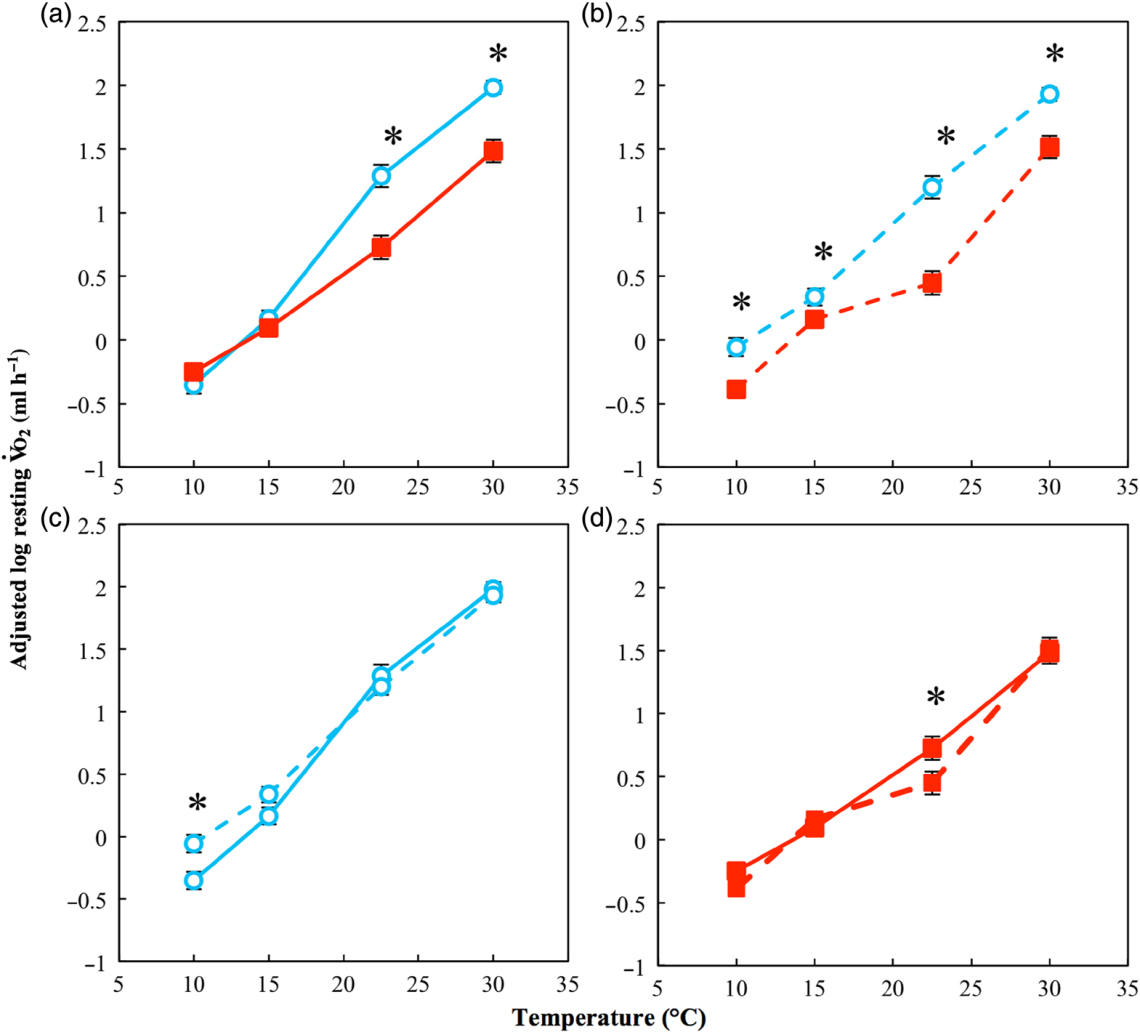
Resting oxygen consumption (V.o_2_rest) of cane toads from low- and high-latitudes acclimated to 15 and 30°C. Filled squares indicate toads acclimated to 30°C; open circles indicate toads acclimated to 15°C. Continuous lines indicate northern populations; dashed lines indicatesouthern populations. (**a**) Low-latitude toads acclimated to 30°C and 15°C; (**b**) high-latitude toads acclimated to 30°C and 15°C; (**c**) low- and high-latitude toads acclimated to 15°C; (**d**) low- and high-latitude toads acclimated to 30°C. Analysis indicates a significant three-way interaction between region, test temperature and acclimation temperature (see Results section for details). For plotting, values of log V.o_2_rest have been adjusted for the scaling effect of log mass. *Statistically significant (*P* < 0.05) pairwise differences at individual temperatures. *n* = 20 for each data point, and error bars represent SEM.

There was a significant positive association between log(V.o_2_rest) and log(mass) ($\chi_{1}^{2}=49.24$, *P* < 0.001), and there was a significant three-way interaction between test temperature, acclimation temperature and region ($\chi_{1}^{2}=7.54$, *P* = 0.006), indicating that between high- and low-latitude regions the effect of acclimation temperature on V.o_2_rest varied with test temperature (Fig. 1). Among low-latitude toads, *post hoc* pairwise analyses showed significantly higher V.o_2_rest for cold-acclimated animals at 22.5 and 30°C, and no difference at 10 and 15°C (Fig. 1a). Among high-latitude toads, *post hoc* pairwise analyses showed significantly higher V.o_2_rest for cold-acclimated animals at 10, 15, 22.5 and 30°C (Fig. 1b). Among all cold-acclimated toads, *post hoc* pairwise analyses showed significantly higher values of V.o_2_rest for high-latitude animals than low-latitude counterparts at 10 and 15°C and no significant difference between 22.5 and 30°C (Fig. 1c). See Supplementary Table S1 for parameter estimates and test statistics for the minimum adequate model used.

### Peak post-exercise rate of oxygen uptake

**Figure 2:**
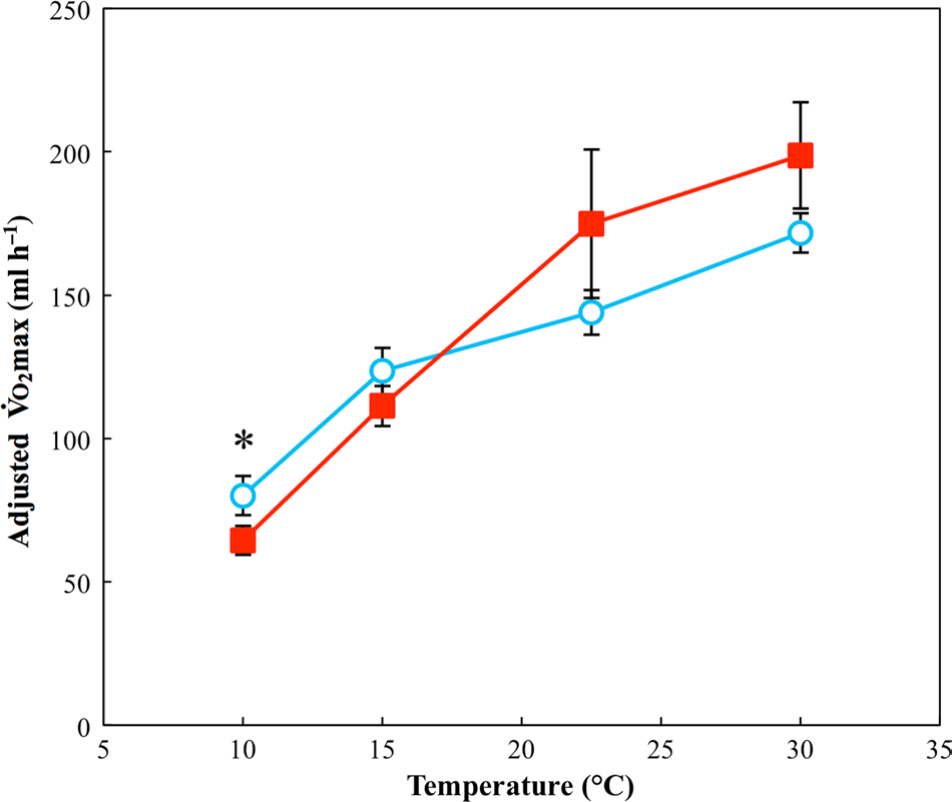
Peak post-exercise rate of oxygen consumption (V.o_2_max) for toads acclimated to 15 and 30°C. Filled squares indicate toads acclimated to 30°C; open circles indicate toads acclimated to 15°C. Data points indicate the combined response of toads from both low- and high-latitudes. Analysis indicates a significant two-way interaction of test temperature and acclimation temperature (see Results section for details). For plotting, values for V.o_2_max have been adjusted for the scaling effect of log mass. *Statistically significant (*P* < 0.05) pairwise differences at individual temperatures. *n* = 40 for the group acclimated to 30°C and *n* = 39 for the group acclimated to 15°C. Error bars represent SEM.

There was a significant positive association between V.o_2_max and log(mass) ($\chi_{1}^{2}=19.4$, *P* < 0.001), and there was a significant two-way interaction between test temperature and acclimation temperature ($\chi_{1}^{2}=6.26$, *P* = 0.012). There was no effect of region, indicating that high- and low-latitude toads exhibit the same response, which is an effect of acclimation temperature that varies with test temperature (Fig. 2). A *post hoc* pairwise analysis showed significantly higher V.o_2_max only at 10°C. See Supplementary Table S2 for parameter estimates and test statistics for the minimum adequate model used.

### Locomotor endurance

For time to exhaustion, there was a significant interaction between test temperature and acclimation temperature ($\chi_{1}^{2}=47.34$, *P* < 0.001), but no effect of log(mass) ($\chi_{1}^{2}=4.24,$*P* = 0.12). Both high- and low-latitude toads showed very similar patterns of endurance, with cold-acclimated animals becoming exhausted in approximately half the time at 22.5 and 30°C (Supplementary Fig. S2). A *post hoc* pairwise analysis showed significantly longer time to fatigue at 22.5 and 30°C, while at 10 and 15°C there was no significant difference. See Supplementary Table S3 for parameter estimates and test statistics for the minimum adequate model used.

### Burst locomotor performance

For jump distance, there was a significant positive association with log(mass) ($\chi_{1}^{2}=32.6$, *P* < 0.001), and there was a significant two-way interaction between test temperature and region ($\chi_{1}^{2}=8.35$, *P* = 0.004), indicating that there was no effect of acclimation temperature, but that the effect of test temperature on performance varied with region (Supplementary Fig. S3). *Post hoc* pairwise analyses showed no significant difference at any test temperature. See Supplementary Table S4 for parameter estimates and test statistics for the minimum adequate model used.

### Aerobic scope

**Figure 3:**
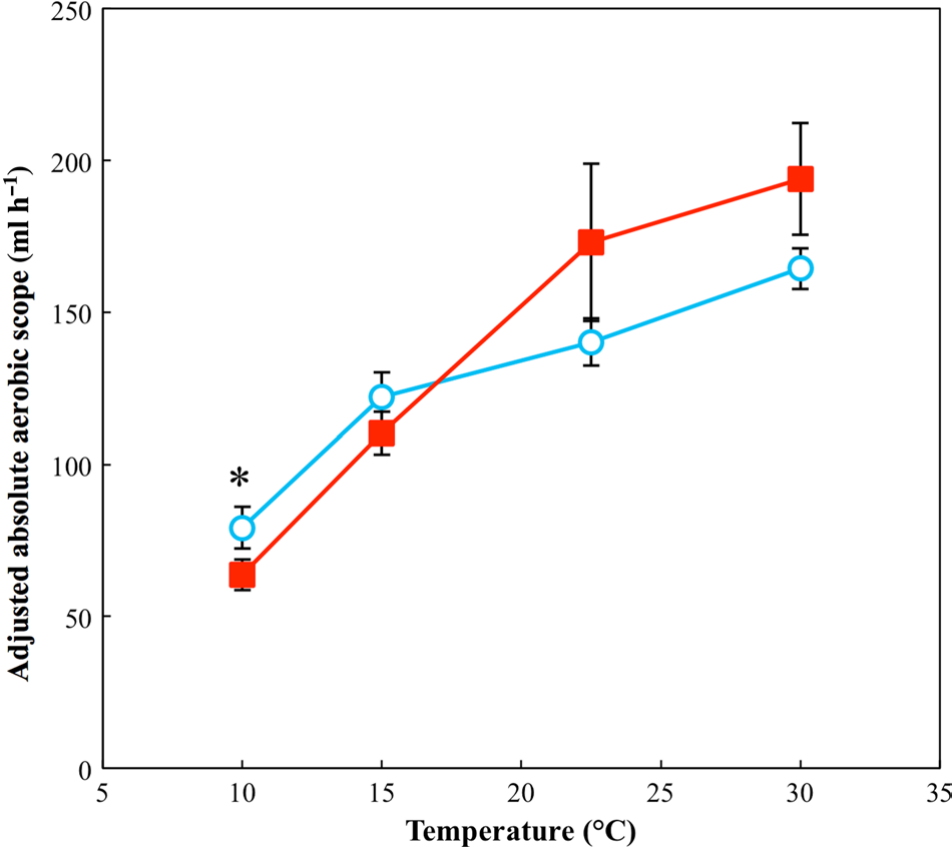
Absolute aerobic scope for toads acclimated to 15 and 30°C. Filled squares indicate toads acclimated to 30°C; open circles indicate toads acclimated to 15°C. Data points indicate combined response of toads from both low- and high-latitudes. For plotting, values were calculated from V.o_2_max and resting V.o_2_ that was adjusted for the scaling effect of body mass. Analysis indicates a significant two-way interaction of test temperature and acclimation temperature (see Results section for details). *Statistically significant (*P* < 0.05) pairwise differences at individual temperatures. *n* = 40 for the group acclimated to 30°C and *n* = 39 for the group acclimated to 15°C. Error bars represent SEM.

There was a significant positive association between absolute aerobic scope and log(mass) ($\chi_{1}^{2}=18.77$, *P* < 0.001) and a significant two-way interaction between test temperature and acclimation temperature ($\chi_{1}^{2}=6.87$, *P* = 0.008). The pattern seen here is almost identical to that of V.o_2_max because values of V.o_2_rest are so small compared with V.o_2_max that they make little difference when subtracted. A *post hoc* analysis showed significantly higher absolute aerobic scope for cold-acclimated toads at 10°C (Fig. 3). See Supplementary Table S5 for parameter estimates and test statistics for the minimum adequate model used.

## Discussion

In the present study, we investigated geographical variation among the physiological traits of V.o_2_rest, V.o_2_max, aerobic scope and locomotor performance. Of the measured traits, only V.o_2_rest and burst locomotor performance showed differences among high- and low-latitude populations. Our most significant finding was that, as hypothesized, cane toads from higher latitudes showed increased thermal metabolic plasticity at low temperature compared with low-latitude counterparts (Fig. 1a, b). As a consequence of this difference in thermal acclimation capacity, high-latitude cane toads have higher resting metabolic rates than low-latitude cane toads when acclimated to cold temperatures (Fig. 1c). This elevation of metabolic rate in low-temperature conditions is likely to benefit high-latitude cane toads through increased rates of ATP production that counteract the thermodynamic depression of critical physiological functions (Seebacher *et al.*, 2009).

The second trait that showed a difference between regions was burst locomotor performance. Within high- and low-latitude populations there was no effect of acclimation temperature, while between regions the pattern observed was the opposite of that which would be expected for a beneficial acclimation response (i.e. where performance is maximized at acclimation temperatures; Supplementary Fig. S3). Other studies have also reported no effect or negative effects of cold acclimation on similar measures of jump performance in a variety of anurans (Miller and Zoghby, 1986; Whitehead *et al.*, 1989; Knowles and Weigl, 1990). As discussed by Wilson and Franklin (2002), if the environmental variable to which the response is expected is in fact stressing or damaging to the animal then it may result in reduced performance. Such negative responses may be trait specific, occurring even as other traits respond favourably (Hoffmann and Hewa-Kapuge, 2000). It is also possible that selection for this trait may vary between high- and low-latitude environments, or perhaps other traits that are more beneficial are selected for at the expense of burst locomotor performance. If two important traits share some common genetic underpinnings, stronger selection for one may have detrimental effects on the other (Leroi *et al.*, 2005).

The theory of OLTT suggests that narrowing aerobic scope may be a limiting factor for ectotherms at the edge of their thermal tolerance (Zielinksi and Pörtner, 1996; De Watchter *et al.*, 1997). Contrary to the predictions of this theory, in cane toads scope is reduced at low temperatures yet the capacity for oxygen uptake following exhaustive exercise remains well in excess of resting metabolic demands (Fig. 3). Likewise, measures of locomotor performance (both sustained and burst) may fail to show an improvement at low temperatures for high-latitude toads because they are simply not important factors limiting the species distribution. While the depressive effects of temperature are certainly clear for all traits measured in this study, if this depression in colder climates does not ultimately impact fitness then there may be no selection pressure for an adaptive change to occur.

The question motivating the present study was whether the continued advance of cane toads in Australia into higher latitudes is the result of, or facilitated by, adaptive changes. The significant differences found in V.o_2_rest between high- and low-latitude cane toads reported here (Fig. 1) provide compelling evidence suggestive of such a change, but the design of the present study is not sufficient to demonstrate that the observed differences have a genetic basis because it only eliminates the effects of relatively short-term acclimation of the order of months. Presumably, the animals collected had lived in their respective environments throughout their lives, and thus the differences detected may be the result of irreversible longer-term acclimation responses or the effect of early development in different thermal environments. The latter has been previously shown to affect acclimation limits in adult zebra fish that underwent embryonic development at different temperatures (Scott and Johnston, 2012). To correct for any such developmental effects, several generations would need to be raised in controlled conditions to obtain test animals divorced from their ancestral environment.

Additionally, inherent in any assertion of adaptive change is the assumption that cane toads from high and low latitudes are migrants from the original introduction in Cairns. This may not be the case. Cane toads were separately introduced into Byron Bay (NSW, Australia) between 1964 and 1966 (van Beurden and Grigg, 1980; Seabrook, 1991). It is not known for certain whether the cane toads introduced into Byron Bay were sourced from Queensland, from their native range or from elsewhere. If high-latitude cane toads do have a different origin or if they are an admixture resulting from separate introductions then they may have genetic differences that were established elsewhere. However, if an origin is shared between regions then adaptation is certainly possible. One prerequisite for genetic differences to arise between populations is sufficient isolation to prevent gene flow from homogenizing genetic variation. Populations can be isolated by geographical or other barriers that physically prevent dispersal, but populations can also be isolated if dispersal and migration distances are sufficiently low that gene flow is not sufficient to prevent genetic differences from arising along a spatial gradient (isolation by distance; Wright, 1943). There is empirical evidence to support the assertion that isolation by distance can occur over relatively short time scales and facilitate local adaptation (Mimura and Aitken, 2007). In comparison to other terrestrial vertebrates, this process of genetic differentiation may occur more rapidly in amphibians because their reliance on standing water bodies limits dispersal and reduces interbreeding between populations over relatively short distances (Hillman *et al.*, 2014). Estoup *et al.* (2004) tested for evidence of isolation by distance for cane toads in Australia by analysing 10 microsatellites in multiple populations along two transects. The first began in North Queensland and extended northwest for ∼900 km, and the second began in Byron Bay and extended southeast for ∼50 km. Along each transect there was evidence of genetic separation between populations, supporting a scenario of migration only between adjacent populations, with multiple founder effects occurring in a directional manner. Despite covering a considerably shorter distance, populations along the southern transect showed a higher degree of genetic separation, indicating successive reductions in genetic diversity (Estoup *et al.*, 2004). Such a pattern could be the result of continual selection for individuals with enhanced survival and reproduction at high latitudes, i.e. selection pressures that favour higher thermal metabolic plasticity and metabolic rates at low temperatures.

The cane toad has already gained a considerable foothold within Australia. While the damage is already done in some regions of the continent, there are areas yet untouched by this species. Management decisions regarding the spread of this species depend critically upon whether it will invade pristine ecosystems further or halt at the currently understood limits of its environmental tolerance. The importance of assessing whether adaptation may facilitate the invasion of regions beyond what has been predicted previously is obvious. Here, we add to the growing body of evidence to suggest that the cane toad is in fact evolving rapidly since its introduction to Australia. If the cane toad exceeds invasion predictions by a significant and unexpected margin then not only will the current impacts upon biodiversity intensify, but new species, as yet unaffected, may be impacted by this uniquely successful invader as predator, prey or competitor. This is particularly important as the cane toad invades higher latitudes and different thermal environments, where it will increasingly coexist with a growing list of native inhabitants. These concerns apply not only to this situation but to all instances of invasive species worldwide and illustrate the importance of assessing adaptation of invaders as they move into novel environments. Such assessments will be valuable in ensuring that appropriate decisions can be made to manage this growing threat to global biodiversity.

## Supplementary material

Supplementary material is available at *Conservation Physiology* online.

## Funding

This research was supported by University of Queensland and ARC funding to C.R.W. and C.E.F.

## Supplementary Material

Supplementary Data
