# Supplementary material for: Does greater thermal plasticity facilitate range expansion of an invasive terrestrial anuran into higher latitudes?
Source: Conserv Physiol. 2015 Mar 13;3(1):cov010. doi: 10.1093/conphys/cov010 (PMC4778455; doi:10.1093/conphys/cov010)
Supplement: Supplementary Data [file cov010supp.zip › cov010supp.docx]

*Statistical model simplification*

To account for the possible effect of morning versus afternoon measurements on V.o_2_rest data, a fixed effect of time of day (AM or PM) was included in the analysis for this trait. Acclimation time and all random effects were non-significant, except for ID (χ^2^_1_ = 5.21, P = 0.022), and the final model used to explain V.o_2_rest was log(V.o_2_rest) ~ test temperature + acclimation temperature + test temperature*acclimation temperature + region + test temperature*region + acclimation temperature*region + test temperature*acclimation temperature*region + log(mass) + ID. The linear models used for post-hoc pairwise comparisons of V.o_2_rest at individual test temperatures within region was log( V.o_2_rest) ~ acclimation temperature + log(mass), and within acclimation temperature, the model used was log( V.o_2_rest) ~ region + log(mass).

To account for the possible effect that the differences in measurement regime between high and low latitude toads may have had on V.o_2_max and locomotor performance data, day was included as a fixed effect whereby low latitude animals had a value of one to four while high latitude animals had a value of one. For V.o_2_max, acclimation time, day, and all random effects were non-significant, except for ID (χ^2^_1_ = 7.23, P = 0.007), and the final model used was V.o_2_max ~ test temperature + acclimation temperature + test temperature*acclimation temperature + region + log(mass) + ID. The linear models used for post-hoc pairwise comparisons of the effect of acclimation temperature at individual test temperatures was V.o_2_max ~ region + log(mass). The model used for locomotor endurance was the same as for V.o_2_max with time to fatigue as the response variable and temperature as a categorical variable due to the non-linearity of the data. ID was the only significant random effect (χ^2^_1_ = 10.99, P < 0.001) but as log(mass) was not found to be significant in the final model, the model for post-hoc pairwise comparisons of endurance at individual test temperatures within acclimation temperature was time to fatigue ~ acclimation temperature.

For the analysis of burst locomotor performance data, to account for the effect of fatigue, the time at which the longest jump was performed during the exercise session was included as a fixed effect. Time of jump, day, acclimation time, and population were non-significant, and there were significant effects of container (χ^2^_1_ = 4.52, P = 0.03) and ID (χ^2^_1_ = 8.42, P = 0.004). The final model used was jump distance ~ test temperature + acclimation temperature + test temperature*region + region + log(mass) + ID + container. For post-hoc pairwise comparisons of the effect of region at individual test temperatures the model used was jump distance ~ region + log(mass) + container.

Analysis of absolute aerobic scope included day as a fixed effect to account for differences in the V.o_2_max measurement regime between high and low latitude toads. Acclimation time, day, and all random effects were non-significant, except for ID (χ^2^_1_ = 7.11, P = 0.007), and the final model used was absolute aerobic scope ~ test temperature + acclimation temperature + test temperature*acclimation temperature + region + log(mass) + ID.

*Tables of estimated effects for minimum adequate models used*

| **Table S1:** Parameter estimates and test statistics for the minimum adequate model used to analyse resting oxygen consumption | | | |
| --- | --- | --- | --- |
|  | Estimate | Std. Error | t value |
| (Intercept) | -5.9972347 | 0.5250799 | -11.422 |
| Ttest | 0.1540864 | 0.0099930 | 15.419 |
| Tacclimation | 0.0270028 | 0.0090279 | 2.991 |
| regionSouth | 1.0297698 | 0.3036102 | 3.392 |
| logMass | 0.8932730 | 0.1088425 | 8.207 |
| Ttest:Tacclimation | -0.0022434 | 0.0004216 | -5.321 |
| Ttest:regionSouth | -0.0399359 | 0.0141293 | -2.826 |
| Tacclimation:regionSouth | -0.0385292 | 0.0127799 | -3.015 |
| Ttest:Tacclimation:regionSouth | 0.0014033 | 0.0005959 | 2.355 |

| **Table S2:** Parameter estimates and test statistics for the minimum adequate model used to analyse peak post-exercise rate of oxygen uptake | | | |
| --- | --- | --- | --- |
|  | Estimate | Std. Error | t value |
| (Intercept) | -456.7376 | 120.104 | -3.803 |
| Ttest15 | 42.8838 | 34.8511 | 1.230 |
| Ttest22.5 | 20.0204 | 34.8501 | 0.574 |
| Ttest30 | 50.4726 | 34.8469 | 1.448 |
| Tacclimation | -1.3363 | 1.1391 | -1.173 |
| regionSouth | -21.2014 | 11.0046 | -1.927 |
| logMass | 127.0884 | 26.7425 | 4.752 |
| Ttest15:Tacclimation | 0.1446 | 1.4638 | 0.099 |
| Ttest22.5:Tacclimation | 3.0757 | 1.4637 | 2.101 |
| Ttest30:Tacclimation | 2.9203 | 1.4637 | 1.995 |

| **Table S3:** Parameter estimates and test statistics for the minimum adequate model used to analyse locomotor endurance | | | |
| --- | --- | --- | --- |
|  | Estimate | Std. Error | t value |
| (Intercept) | -2.968e+02 | 3.858e+02 | -0.769 |
| Ttest15 | 1.122e+02 | 1.143e+02 | 0.982 |
| Ttest22.5 | -1.410e+02 | 1.143e+02 | -1.234 |
| Ttest30 | -2.426e+01 | 1.144e+02 | -0.212 |
| Tacclimation | 4.624e-01 | 3.705e+00 | 0.125 |
| regionSouth | -3.253e+01 | 3.534e+01 | -0.920 |
| logMass | 8.930e+01 | 8.586e+01 | 1.040 |
| Ttest15:Tacclimation | 7.438e-03 | 4.800e+00 | 0.002 |
| Ttest22.5:Tacclimation | 2.880e+01 | 4.800e+00 | 6.001 |
| Ttest30:Tacclimation | 1.789e+01 | 4.817e+00 | 3.714 |

| **Table S4:** Parameter estimates and test statistics for the minimum adequate model used to analyse burst locomotor performance | | | |
| --- | --- | --- | --- |
|  | Estimate | Std. Error | t value |
| (Intercept) | -10.57876 | 4.60817 | -2.296 |
| Ttest | 0.26846 | 0.02762 | 9.720 |
| Tacclimation | -0.01471 | 0.03862 | -0.381 |
| regionSouth | -2.82198 | 0.95527 | -2.954 |
| logMass | 6.91562 | 1.02299 | 6.760 |
| Ttest:regionSouth | 0.09905 | 0.03866 | 2.562 |

| **Table S5:** Parameter estimates and test statistics for the minimum adequate model used to analyse absolute aerobic scope | | | |
| --- | --- | --- | --- |
|  | Estimate | Std. Error | t value |
| (Intercept) | -445.9157 | 119.8517 | -3.721 |
| Ttest15 | 42.2637 | 34.7956 | 1.215 |
| Ttest22.5 | 15.3965 | 34.7946 | 0.442 |
| Ttest30 | 41.7629 | 34.7914 | 1.200 |
| Tacclimation | -1.3230 | 1.1370 | -1.164 |
| regionSouth | -21.1055 | 10.9811 | -1.922 |
| logMass | 124.4181 | 26.6858 | 4.662 |
| Ttest15:Tacclimation | 0.1488 | 1.4615 | 0.102 |
| Ttest22.5:Tacclimation | 3.1889 | 1.4613 | 2.182 |
| Ttest30:Tacclimation | 3.0730 | 1.4614 | 2.103 |

*Supplementary Figure Legends*

**Figure S1.** Locations from which cane toads were collected with mean monthly maximum and minimum temperature data from 2001 to 2011. Solid lines = mean maximum temperature and dashed lines = mean minimum temperature. G = Gulf, coordinates and data from Kowanyama Airport. M = Mareeba, coordinates and data from Mareeba Airport. B = Ballina, coordinates and data from Ballina Airport. Y = Yamba, coordinates and data from Yamba pilot station. Coordinates and temperature data obtained from Australian Bureau of Meteorology ([www.bom.gov.au](http://www.bom.gov.au)).

**Figure S2.** Time to fatigue for toads acclimated to 15°C and 30°C. Solid square markers = 30°C acclimated toads, open circle markers = 15°C acclimated toads. Data points indicate combined response of toads from both northern and southern regions. Analysis indicates a significant two way interaction of test temperature and acclimation temperature (see *Results* for details). ***** indicates statistically significant (p < 0.05) pair-wise differences at individual temperatures. N = 39 for all data points and error bars = s.e.m.

**Figure S3.** Jump distance for toads from northern and southern regions. Solid square markers = northern toads, open circle markers = southern toads. There was no effect of acclimation, so points are pooled data for toads from 15 and 30°C acclimation groups. Analysis indicates a significant two-way interaction of region and test temperature (see *Results* for details). For plotting, values for jump distance have been adjusted for the scaling effect of log mass. N = 39 for all data points. Error bars = s.e.m.
