# Supplementary figures and images for: Does greater thermal plasticity facilitate range expansion of an invasive terrestrial anuran into higher latitudes?
Source: Conserv Physiol. 2015 Mar 13;3(1):cov010. doi: 10.1093/conphys/cov010 (PMC4778455; doi:10.1093/conphys/cov010)

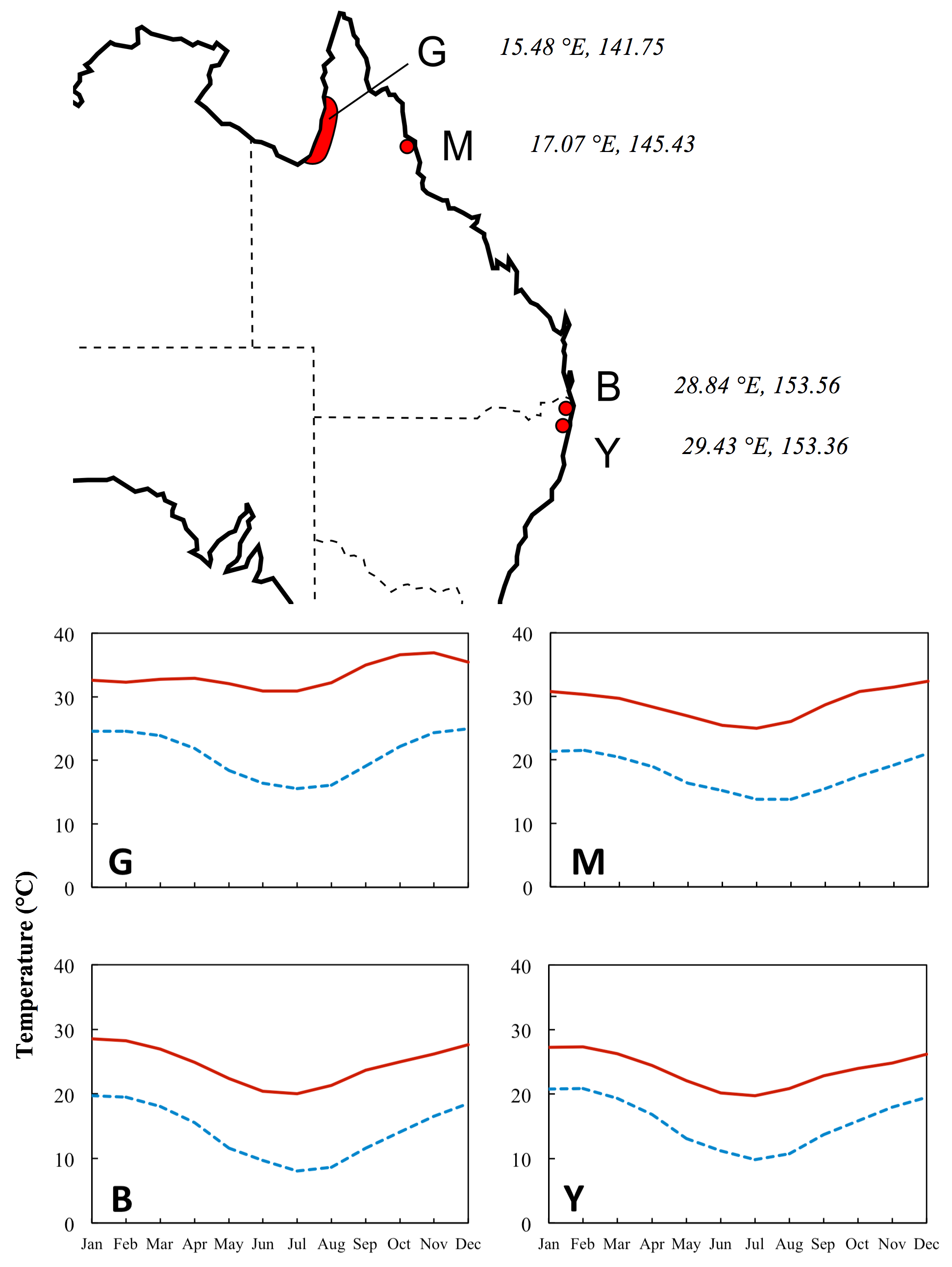

Supplement: Supplementary Data [file cov010supp.zip › cov010supp_fig1.tif]

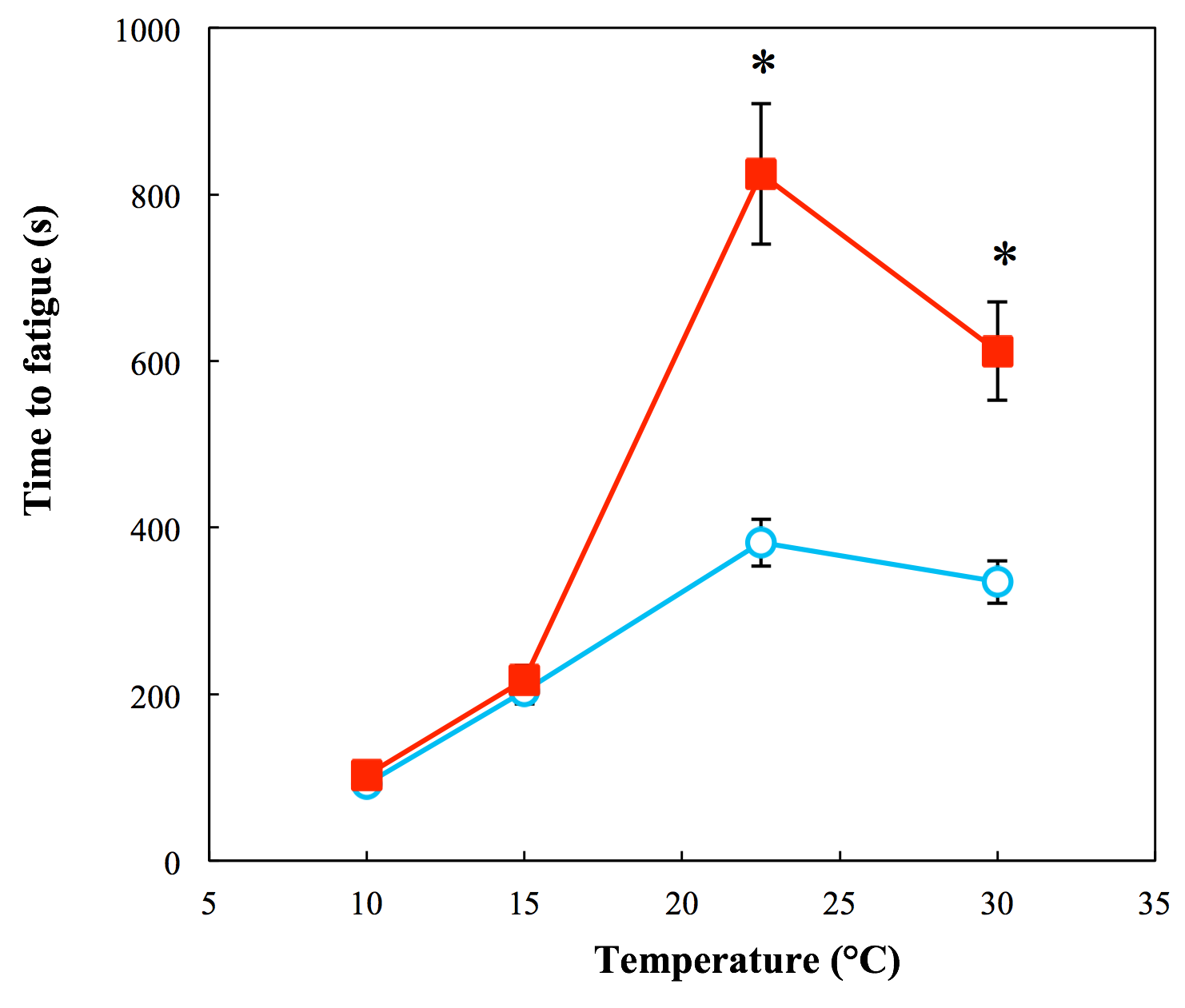

Supplement: Supplementary Data [file cov010supp.zip › cov010supp_fig2.tif]

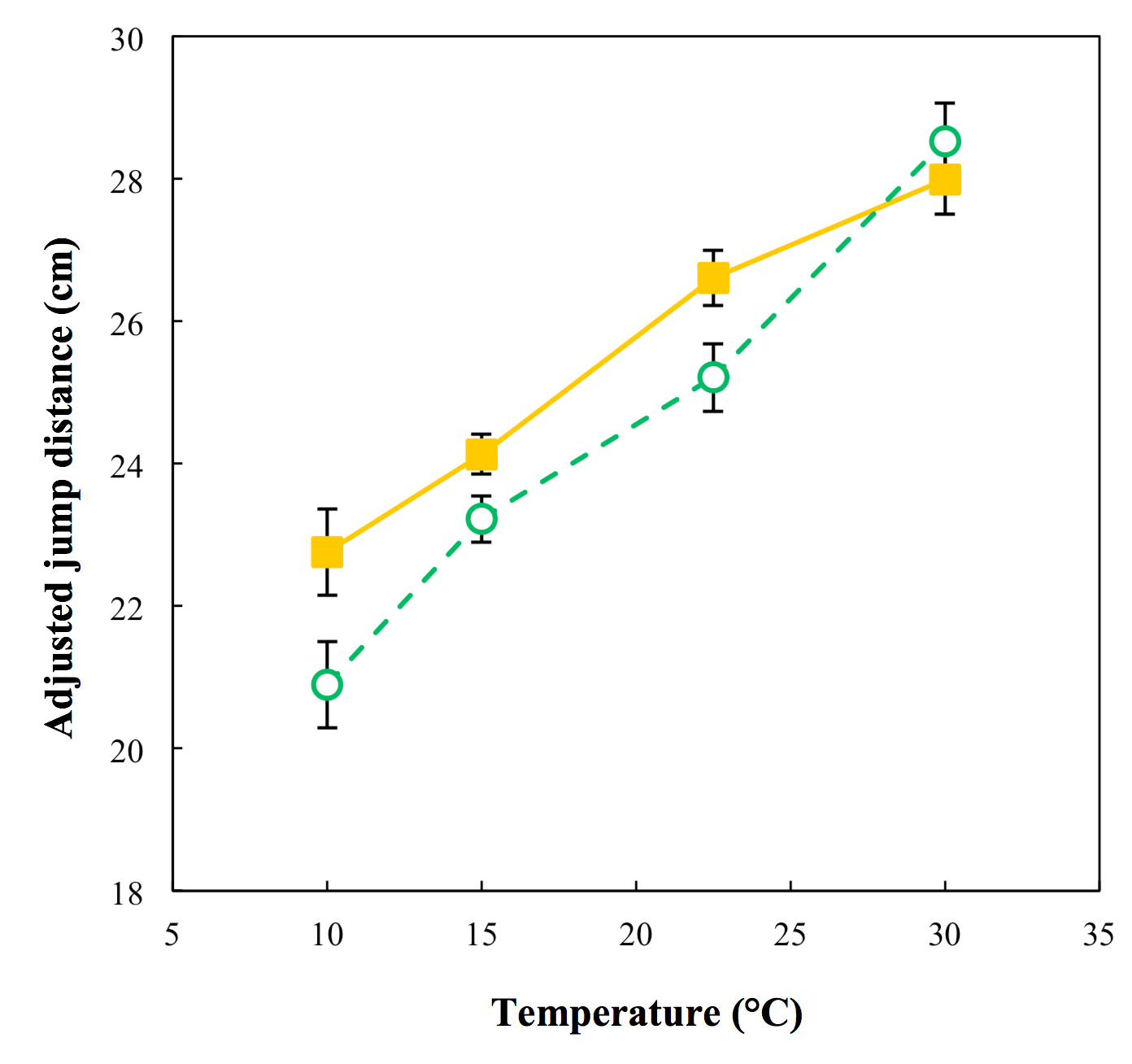

Supplement: Supplementary Data [file cov010supp.zip › cov010supp_fig3.tif]
